# Sex differences in the association between sleep duration and frailty in older adults: evidence from the KNHANES study

**DOI:** 10.1186/s12877-024-05004-2

**Published:** 2024-05-16

**Authors:** Beomman Ha, Mijin Han, Wi-Young So, Seonho Kim

**Affiliations:** 1 Armed Forces Medical Command, Seongnam-si, 13574 Republic of Korea; 2 Chungbuk National University Hospital, Cheongju-si, 28644 Republic of Korea; 3 Department of Sports Medicine, College of Humanities, Korea National University of Transportation, 50 Daehak-ro, Chungbuk, Chungju-si, 27469 Republic of Korea; 4 Department of Nursing and Research Institute of Nursing Science, College of Medicine, Chungbuk National University, Cheongju-si, 28644 Republic of Korea

**Keywords:** Sleep duration, Frailty, Older adults, Sex differences

## Abstract

**Background:**

Frailty is a pervasive clinical syndrome among the older population. It is associated with an increased risk of diverse adverse health outcomes including death. The association between sleep duration and frailty remains unclear. Therefore, the aim of this study was to investigate the relationship between sleep duration and frailty in community-dwelling Korean older adults and to determine whether this relationship is sex-dependent.

**Methods:**

Data on 3,953 older adults aged *≥* 65 years were obtained from the 7th (2016–2018) Korea National Health and Nutrition Examination Survey (KNHANES). Frailty was defined using the Fried phenotype with criteria customized for the KNHANES dataset. Self-reported sleep duration was classified as short sleep duration (≤ 6 h), middle sleep duration (6.1–8.9 h), and long sleep duration (≥ 9 h). Complex samples multivariate logistic regression analysis was conducted to estimate odds ratios (ORs) and 95% confidence intervals (CIs).

**Results:**

The percentage of male participants with short, middle, and long sleep durations was 34.9%, 62.1%, and 16.8%, respectively, while that of female participants was 26.1%, 59.2%, and 14.7%. The prevalence of frailty in the middle sleep duration group was lower than that in the short and long sleep duration groups in both men (short, 14.7%; middle, 14.2%; long, 24.5%; *p* < 0.001) and women (short, 42.9%; middle, 27.6%; long, 48.6%; *p* < 0.001). Both short (OR = 2.61, 95% CI = 1.91 − 4.83) and long (OR = 2.57, 95% CI = 1.36 − 3.88) sleep duration groups had a significantly higher OR for frailty than the middle sleep duration group even after adjusting for confounding variables among women, but not among men.

**Conclusion:**

Short and long sleep durations were independently associated with frailty in community-dwelling Korean older adult women. Managing sleep problems among women should be prioritized, and effective interventions to prevent frailty should be developed accordingly.

## Background

Frailty is a state of increased vulnerability to stressors that results from decreased physiological reserves and multisystem dysregulation, or a state of limited capacity to maintain homeostasis and respond to internal and external stresses [1]. In community-dwelling older adults, frailty is associated with an increased risk of adverse health outcomes including falls, disability, hospitalization, institutionalization, and death [2, 3].

The prevalence of frailty among community-dwelling older adults varies widely depending on age, sex, and country. Although direct comparisons may be limited because of factors such as differences in frailty assessment methods, the prevalence of frailty in Korea (23.1%) is relatively higher than that in other countries such as the United States (7.1%), Japan (7.4%) and China (10.1%) [4–7]. Although the cause of frailty has not been fully identified yet, its prevalence increases with age. By 2050, the proportion of people aged *≥* 65 years in Korea is predicted to be 39.8% [8]. Frailty is an important public health issue for older adults in the aging Korean population owing to its prevalence and social burden. However, frailty is partially reversible and preventable [9, 10]. Therefore, a better understanding of the risk factors associated with frailty is crucial to develop new strategies for its prevention and management.

Appropriate sleep is crucial to the overall health and well-being of older adults. However, sleep disturbance is reportedly experienced by approximately 50% of this population [11, 12]. In Korea, 32.4% of older adults in the Korean community experience sleep disorders [13], and 61.8% do not sleep for the duration recommended by The National Sleep Foundation [14]. In addition, older adults with sleep problems may have a lower quality of life, cognitive decline, depression, and other adverse health outcomes including frailty [15].

Sleep disturbances and frailty often coexist and are interrelated, especially among older adults [16], and recent studies have shown that frailty is significantly associated with sleep duration among these individuals [17, 18]. While some studies [17, 19–22] have reported that long sleep duration was associated with a greater risk of frailty, others [23, 24] reported that short sleep duration was associated with frailty. However, some studies [21, 25] found that both long and short sleep durations were associated with frailty. In addition, the relationship between sleep duration and frailty is reportedly sex-dependent [5, 20, 23, 24]. Investigation of the relationship between sleep duration and frailty is crucial and essential for managing sleep problems and frailty among the Korean older adults. Nevertheless, only few studies have investigated this relationship community-dwelling Korean older adults [14, 20]. Therefore, this study aimed to examine the relationship between sleep duration and frailty in community-dwelling Korean older adults and to determine whether this association is sex-dependent. Based on existing literature, we hypothesized that individuals with a short or long sleep duration exhibit more severe frailty than those with a normal sleep duration among Korean older adults, and there are sex differences in the association between sleep duration and frailty after controlling for sociodemographic variables, lifestyle-related variables, and health-related variables.

## Methods

### Study design and participants

This study used data from the 7th (2016 − 2018) Korea National Health and Nutrition Examination Survey (KNHANES). The KNHANES, conducted annually by the Korea Center for Disease Control (KCDC) and the Korean Ministry of Health and Welfare, is a nationwide population-based survey designed to assess the health-related behavior, health conditions, and nutritional status of Korean adults. The survey uses stratified, multistage, clustered probability sampling methods to select a representative sample of civilian non-institutionalized Korean adults (the plan, protocol, and survey license are available at http://knhanes.cdc.go.kr). The inclusion criteria for the current study were as follows: age *≥* 65 years and presence of relevant data. Of the 24,269 participants from the 7th KNHANES, we included 3,953 participants in the current analysis after excluding 19,313 participants aged < 65 years and 1,003 with missing data on frailty, sleep duration, and covariate variables. All participants provided written informed consent, and the survey collection process was approved by the KCDC Research Ethics Review Committee (Institutional Review Board number: 2018-01-03-P-A).

### Assessment of sleep duration

The participants were queried about their usual times for the onset of sleep and waking. Self-reported sleep duration was calculated as the difference between these times. As previously described [18, 23], we classified participants into three groups according to their self-reported sleep duration: short ≤ 6 h, middle 6.1–8.9 h, and long ≥ 9 h.

### Assessment of frailty

The outcome variable in this study was frailty, assessed as per a modified frailty phenotype developed by Fried et al. [3]: (1) unintended weight loss (self-reported; ≥3 kg in the past year) [26], (2) weakness (handgrip strength < 26 kg for men and < 18 kg for women based on the Asian Working Group for Sarcopenia 2014) [27], (3) slowness (responded “I have slight difficulty walking” or “I have to stay in the bed all day” in at least one of two questions in the Euro Quality of life 5-Dimensions, a motor ability indicator) [28], (4) emotional exhaustion (responded “I feel considerably exhausted” to the level of stress awareness question) [29], and (5) low physical activity (< 2 h of moderate-intensity physical activity/week or < 1 h of high-intensity physical activity/week) [30]. Participants who met three or more criteria were classified as “frail,” one or two as “pre-frail,” and zero as “robust.”

### Covariates

Sociodemographic characteristics included sex (man/woman), age (65 − 69/70 − 74/75 − 79/≥80 years), education level (≤ 6 years/≥7 years), household income (lower/lower middle/upper middle/upper), living arrangement (alone/with family), and area of residence (urban/rural). Lifestyle-related variables included self-rated health status (good/poor), current smoking (yes/no), and current drinking (yes/no). Health-related variables included the number of chronic diseases (0/1 − 2/≥3), body mass index (BMI) (underweight/normal weight/overweight/obese), pain (yes/no), and limitation of daily life (yes/no). Chronic diseases included hypertension, stroke, myocardial infarction, angina, osteoarthritis, rheumatoid arthritis, asthma, diabetes mellitus, cancer (stomach, liver, colon, breast, cervical, and lung), and liver disease (hepatitis B, hepatitis C, and cirrhosis). BMI was calculated as weight (kg) divided by the square of the height (m^2^) and defined (according to the Korean guidelines and KNHANES recommendations) as follows: underweight, < 18.5 kg/m^2^; normal weight, 18.5–22.9 kg/m^2^; overweight, 23–24.9 kg/m^2^; and obese, ≥ 25 kg/m^2^ [31]. Limitation of daily life was evaluated using a questionnaire focused on daily life and social activities affected by health problems, physical disorders, or mental disorders.

### Statistical analyses

Statistical analyses were performed using IBM SPSS (version 26.0; IBM Corp., Armonk, NY, USA). Based on statistical guidance from the KCDC, we used a complex sample analysis method to utilize all weighted data from the KNHANES. Descriptive statistics were calculated as frequencies and percentages. The Chi-square test was used to compare categorical variables among groups. Complex samples multivariate logistic regression analysis was used to estimate the odds ratios (ORs) and 95% confidence intervals (CIs) for the association between sleep duration and frailty. Potential confounding factors, including age, education level, household income, living arrangements, area of residence, self-rated health status, smoking status, drinking status, number of chronic diseases, BMI, pain, and limitation of daily life, were adjusted for. Due to the significant sex differences in frailty, data from men and women were analyzed separately. Statistical significance was defined as *p* < 0.05.

## Results

The characteristics of the participants according to sleep duration stratified by sex are presented in Table 1. Among men, there were 375 (34.9%), 1,104 (62.1%), and 299 (16.8%) participants in the short, middle, and long sleep duration groups, respectively. All covariates differed among the three groups of different sleep durations, except for current smoking and the number of chronic diseases. While in women, there were 568 (26.1%), 1, 287 (59.2%), and 320 (14.7%) participants in the short, middle, and long sleep duration groups, respectively. There were differences in age, education, household income, area of residence, self-rated health, and limitation of daily life among the sleep duration groups. The prevalence rate of frailty in the middle sleep duration group was lower than that in the short and long sleep duration groups in both men (short, 14.7%; middle, 14.2%; long, 24.5%; *p* < 0.001) and women (short, 42.9%; middle, 27.6%; long, 48.6%; *p* < 0.001). These results were similar in each frailty component, except for unintended weight loss (Table 1).

**Table 1:** Characteristics of participants according to sleep duration stratified by sex (*N* = 3953)

Variables	Men (*n* = 1778)				Women (*n* = 2175)			
	Short (*n* = 375)	Middle (*n* = 1104)	Long (*n* = 299)	*p*	Short (*n* = 568)	Middle (*n* = 1287)	Long (*n* = 320)	*p*
Age group, years								
65 − 69	155 (46.8)	371 (36.7)	81 (29.6)	< 0.001	185 (30.6)	474 (34.9)	67 (19.0)	< 0.001
70 − 74	109 (27.7)	325 (28.6)	74 (24.1)		139 (21.5)	363 (25.7)	76 (23.2)	
75 − 79	69 (14.8)	249 (20.9)	82 (26.3)		142 (28.6)	285 (24.7)	81 (26.2)	
≥ 80	42 (10.7)	159 (18.3)	62 (20.0)		102 (19.3)	165 (14.6)	96 (31.6)	
Education ≤ 6 years	139 (36.8)	399 (34.1)	162 (52.4)	< 0.001	398 (71.2)	885 (68.4)	272 (83.9)	< 0.001
Household income								
Lower	145 (37.8)	409 (36.2)	157 (50.7)	0.007	305 (54.9)	625 (48.2)	191 (60.5)	0.012
Lower middle	101 (28.1)	345 (30.6)	84 2(7.6)		137 (22.8)	348 (26.6)	68 (20.3)	
Upper middle	72 (19.1)	204 (19.1)	36 (13.2)		77 (13.6)	184 (18.7)	32 (8.9)	
Upper	57 (15.0)	146 (14.1)	22 (8.5)		49 (8.7)	130 (10.5)	29 (10.3)	
Living arrangement, alone	53 (13.1)	133 (10.5)	39 (12.2)	0.041	185 (29.9)	375 (25.4)	93 (26.5)	0.116
Area of residence, urban	286 (80.1)	837 (80.2)	191 (67.7)	< 0.001	441 (82.1)	948 (78.0)	198 (64.9)	< 0.001
Self-rated health, good	285 (77.6)	852 (78.1)	204 (68.1)	0.007	346 (61.2)	867 (67.9)	184 (58.4)	0.004
Current smoking, yes	72 (18.3)	184 (17.4)	58 (18.8)	0.873	15 (2.4)	20 (1.6)	6 (1.3)	0.366
Current drinking, yes	265 (72.9)	754 (69.7)	183 (62.0)	0.031	224 (38.3)	515 (39.6)	113 (34.4)	0.323
No. of chronic disease(s)^a^								
0	99 (25.3)	309 (28.7)	69 (22.0)	0.109	99 (18.0)	246 (19.1)	60 (20.1)	0.866
1 − 2	239 (65.2)	692 (62.4)	190 (64.4)		371 (65.2)	829 (63.6)	211 (65.1)	
≥ 3	37 (9.5)	103 (8.8)	40 (13.6)		98 (16.8)	212 (17.2)	49 (14.8)	
Body mass index								
Underweight	15 (3.5)	30 (2.9)	8 (2.8)	0.024	13 (2.9)	25 (2.1)	7 (2.1)	0.834
Normal weight	143 (37.3)	495 (44.9)	162 (53.8)		216 (39.7)	501 (38.9)	117 (36.9)	
Overweight	120 (32.3)	324 (29.9)	78 (26.8)		172 (29.3)	384 (29.0)	104 (33.0)	
Obese	97 (26.9)	255 (22.3)	51 (16.6)		167 (28.1)	377 (29.9)	92 (27.9)	
Pain, yes	116 (29.0)	255 (21.5)	101 (35.2)	< 0.001	274 (46.9)	539 (41.0)	144 (44.6)	0.137
Limitation of daily life, yes	54 (12.2)	140 (11.8)	69 (21.9)	< 0.001	107 (18.2)	249 (18.3)	97 (30.1)	< 0.001
Frailty status, frailty	60 (14.7)	156 (14.2)	75 (24.5)	< 0.001	229 (42.9)	418 (27.6)	143 (48.6)	< 0.001
Components of frailty, yes								
Unintended weight loss	62 (15.6)	150 (13.6)	48 (15.4)	0.617	83 (14.5)	179 (14.7)	48 (15.5)	0.930
Weakness	74 (18.3)	272 (23.6)	129 (40.2)	< 0.001	321 (56.9)	645 (49.2)	217 (65.5)	< 0.001
Slowness	104 (26.4)	276 (23.7)	103 (32.1)	0.042	269 (45.7)	528 (40.6)	168 (48.0)	0.044
Exhaustion	52 (12.6)	119 (11.0)	50 (16.2)	0.069	154 (26.7)	284 (21.4)	81 (24.4)	0.034
Low physical activity	240 (62.0)	698 (60.5)	210 (66.6)	0.459	424 (73.1)	901 (70.2)	285 (86.3)	< 0.001

Notes: Uncounted n (weighted %), Sleep duration was classified as short(≤ 6 h), middle (6.1–8.9 h) or long (≥ 9 h). The chi-square test was used to determine *p*-values

^a^Chronic diseases include hypertension, stroke, myocardial infarction, angina, osteoarthritis, rheumatoid arthritis, asthma, diabetes mellitus, cancer, and liver disease

The characteristics of the participants according to frailty status stratified by sex are presented in Table 2. Among men, there were 346 (19.4%), 1,141 (64.1%), and 291 (16.3%) participants in the robust, pre-frailty, and frailty groups, respectively, while in women, there were 188 (8.6%), 1,197 (55.0%), and 790 (36.3%). In men, all covariates differed among the three groups. In women, all covariates, except for the living arrangement, current smoking, and BMI, differed among the three groups (Table 2).

**Table 2:** Characteristics of participants according to frailty status stratified by sex (*N* = 3953)

Variables	Men (*n* = 1778)				Women (*n* = 2175)			
	Robust (*n* = 346)	Pre-frailty (*n* = 1141)	Frailty (*n* = 291)	*p*	Robust (*n* = 188)	Pre-frailty (*n* = 1197)	Frailty (*n* = 790)	*p*
Age group, years								
65 − 69	166 (51.1)	391 (37.2)	50 (22.4)	< 0.001	104 (55.9)	451 (35.7)	171 (20.0)	< 0.001
70 − 74	113 (31.7)	336 (28.5)	59 (18.7)		60 (31.9)	334 (25.3)	184 (20.9)	
75 − 79	47 (11.6)	259 (21.2)	94 (29.1)		18 (8.8)	268 (25.5)	222 (30.6)	
≥ 80	20 (5.6)	155 (13.1)	88 (29.8)		6 (3.4)	144 (13.5)	213 (28.5)	
Education ≤ 6 years	79 (20.2)	472 (40.3)	149 (50.6)	< 0.001	88 (45.1)	807 (66.7)	660 (84.0)	< 0.001
Household income								
Lower	90 (26.2)	433 (37.1)	188 (63.7)	< 0.001	68 (35.1)	567 (47.5)	486 (61.6)	< 0.001
Lower middle	110 (32.0)	358 (30.6)	62 (21.8)		61 (33.1)	320 (25.9)	172 (21.1)	
Upper middle	76 (23.3)	207 (18.6)	29 (9.2)		44 (23.0)	166 (13.9)	83 (11.1)	
Upper	70 (18.5)	143 (13.7)	12 (5.3)		15 (8.7)	144 (12.6)	49 (6.2)	
Living arrangement, alone	36 (8.9)	140 (10.7)	49 (17.2)	0.005	54 (25.7)	340 (25.2)	259 (29.2)	0.190
Area of residence, urban	303 (89.3)	823 (76.6)	188 (69.3)	< 0.001	167 (92.1)	891 (79.0)	529 (71.4)	< 0.001
Self-rated health, good	309 (88.2)	883 (78.6)	149 (51.0)	< 0.001	167 (89.4)	880 (74.0)	350 (45.5)	< 0.001
Current smoking, yes	38 (11.8)	209 (18.6)	67 (22.7)	0.004	3 (1.4)	20 (1.4)	18 (2.4)	0.222
Current drinking, yes	274 (78.7)	768 (69.2)	160 (56.1)	< 0.001	95 (51.6)	502 (40.9)	255 (32.1)	< 0.001
No. of chronic disease(s)^a^								
0	107 (32.1)	311 (26.7)	59 (20.3)	0.001	63 (33.4)	240 (21.5)	102 (11.8)	< 0.001
1 − 2	217 (61.0)	723 (64.2)	181 (63.3)		115 (61.5)	782 (63.4)	514 (66.2)	
≥ 3	22 (6.9)	107 (9.1)	51 (16.5)		10 (5.1)	175 (15.1)	174 (22.0)	
Body mass index								
Underweight	3 (1.1)	21 (1.7)	29 (10.8)	< 0.001	2 (1.0)	23 (2.3)	20 (2.7)	0.074
Normal weight	164 (47.3)	497 (43.3)	139 (47.6)		79 (43.1)	451 (38.8)	304 (38.1)	
Overweight	101 (29.2)	347 (31.3)	74 (25.2)		57 (30.4)	392 (31.9)	211 (26.1)	
Obese	78 (22.4)	276 (23.7)	49 (16.4)		50 (25.5)	331 (27.1)	255 (33.1)	
Pain, yes	36 (10.4)	272 (23.5)	164 (53.4)	< 0.001	31 (16.6)	423 (34.1)	503 (62.5)	< 0.001
Limitation of daily life, yes	21 (7.1)	146 (11.4)	96 (31.3)	< 0.001	10 (4.8)	175 (13.6)	268 (32.7)	< 0.001
Sleep duration								
Short (≤ 6 h)	73 (22.7)	242 (22.3)	60 (14.7)	< 0.001	53 (28.3)	286 (24.2)	229 (42.9)	< 0.001
Middle (6.1–8.9 h)	238 (67.1)	710 (60.6)	156 (14.2)		123 (66.1)	746 (62.6)	418 (27.6)	
Long (≥ 9 h)	35 (10.3)	189 (17.1)	75 (24.5)		12 (5.5)	165 (13.2)	143 (48.6)	

Notes: Uncounted n (weighted %), The chi-square test was used to determine *p*-values

^a^Chronic diseases include hypertension, stroke, myocardial infarction, angina, osteoarthritis, rheumatoid arthritis, asthma, diabetes mellitus, cancer, and liver disease

The associations of sleep duration with frailty and components of frailty were analyzed using complex samples multivariate logistic regression, after the control of age, education level, household income, living arrangement, area of residence, self-reported health status, smoking status, drinking status, number of chronic diseases, BMI, pain, and limitation of daily life. Multivariate analysis revealed that compared with the middle sleep duration group, the odds ratio (OR) for frailty were significantly higher in the short (OR: 2.61, 95% CI: 1.91 − 4.83) and long (OR: 2.57, 95% CI: 1.36 − 3.88) sleep duration groups among women. This association was not observed in men.

According to each component, the long sleep duration group had a significantly higher OR for weakness (OR 1.62, 95% CI 1.24 − 1.97) than the middle sleep duration group among men. In women, compared with the middle sleep duration group, the ORs for weakness (OR 1.95, 95% CI 1.58 − 2.25) and exhaustion (OR 1.34, 95% CI 1.02 − 1.78) were higher in the short sleep duration group, while the ORs for weakness (OR 2.38, 95% CI 1.92 − 2.87) and low physical activity (OR 2.47, 95% CI 1.36 − 3.09) were higher in the long sleep duration group (Table 3).

**Table 3:** Associations of sleep duration with frailty and components of frailty by sex (*N* = 3953)

	Frailty OR (95%CI)	Components of Frailty				
		Weight loss OR (95%CI)	Weakness OR (95%CI)	Slowness OR (95%CI)	Exhaustion OR (95%CI)	Low PA OR (95%CI)
Men						
Middle	1.00	1.00	1.00	1.00	1.00	1.00
Short	1.02 (0.61–1.71)	0.91 (0.61–1.35)	1.26 (0.87–1.84)	0.97 (0.67–1.42)	0.98 (0.65–1.48)	0.99 (0.74–1.30)
Long	1.30 (0.71–2.37)	1.22 (0.76–1.97)	**1.62 (1.24–1.97)** ^*^	1.27 (0.84–1.92)	0.77 (0.49–1.22)	1.07 (0.77–1.49)
Women						
Middle	1.00	1.00	1.00	1.00	1.00	1.00
Short	**2.61 (1.91–4.83)** ^*^	1.15 (0.84–1.57)	**1.95 (1.58–2.25)** ^*^	1.12 (0.86–1.47)	**1.34 (1.02–1.78)** ^*^	1.23 (0.86–1.48)
Long	**2.57 (1.36–3.88)** ^*^	1.17 (0.75–1.82)	**2.38 (1.92–2.87)** ^*^	1.09 (0.62–1.32)	1.04 (0.74–1.47)	**2.47 (1.36–3.09)** ^*^

OR = Odds ratio, CI = Confidence interval, PA = Physical activity

Notes: Multivariabe logistic regression was performed after adjustment for age, educational level, household income, living arrangement, area of residence, self-rated health, current smoking and drinking status, number of chronic diseases, BMI, pain, and limitation of daily life

Sleep duration was classified as short (≤ 6 h), middle (6.1–8.9 h) or long (≥ 9 h)

The odds ratio of frailty was compared against the robust group. *Indicates a significant association

## Discussion

The findings of this study showed that both short and long sleep durations were associated with frailty among women, but not among men. Particularly, short and long sleep durations had a higher OR for frailty than middle sleep duration, even in multivariate analysis after adjusting for age, education level, household income, living arrangement, area of residence, self-rated health status, smoking status, drinking status, number of chronic diseases, BMI, pain, and limitation of daily life.

Literature reports on the relationship between sleep duration and frailty, however, are inconsistent. A previous study [18] on 9,824 Japanese older adults aged 65 and above showed that both short (< 6 h) and long (*≥* 9 h) sleep durations were associated with a higher frailty risk compared to middle sleep duration, extending to statistical analysis stratified by sex. A longitudinal study on Mexican older adults aged 70 and above showed that both short (< 6 h) and long (*≥* 9 h) sleep durations were associated with frailty [21]. In another study, only short sleep duration (< 5 h or < 6 h) was associated with frailty among women, but not among men [23, 32]. On the contrary, other studies found that only long sleep duration (*≥* 9 h or *≥* 10 h) was associated with frailty [19, 20, 22]. Kang et al. [20] reported that long sleep duration (*≥* 8 h), but not short sleep duration (< 6 h), effectively explained frailty in older adult women aged 70 years or older in Korea. In addition, no association was observed between sleep duration and frailty in men. Interestingly, Zhang et al. [5] reported that long sleep duration (≥ 9 h) was associated with frailty in men, while short sleep duration (< 6 h) was associated with frailty in women, this indicated a sex-specific association.

These inconsistencies may be attributed to differences in the frailty measures, sleep duration assessment methods (self-reported vs. actigraphy), or sleep duration classifications (short sleep duration defined as < 5 h, < 6 h, or < 7 h), or due to sex disparity [19, 33]. The inconclusive results of these studies also raise the question of whether older adults in different countries or regions have different sleep requirements [22]. Disruptions in neuroendocrine regulation leading to chronic inflammation, lowered testosterone levels, increased oxidative stress, and growth hormone imbalance may underlie the association between short sleep duration and frailty [34, 35]. Although the exact mechanisms remain unclear, several similar mechanisms underlie the association between long sleep duration and frailty. First, long sleep duration is associated with poor health and reduced physical function [36, 37], which increases frailty risk [38, 39]. Second, people with long sleep durations have high levels of pro-inflammatory factors, such as C-reactive protein (CRP) and interleukin-6 [40], which are positively associated with frailty [41]. In addition, elevated melatonin and cortisol levels, lower body temperature, and immune system imbalances have also been suggested to lead to frailty [42].

Previous studies have reported relationships between sleep duration and frailty components. Our study showed that the prevalence of weakness, slowness, fatigue, and physical inactivity (excluding unintended weight loss) was higher in individuals with short or long sleep duration than in to those with middle sleep duration. The results were similar for women, but for men, only weakness and slowness were statistically significant. Previous studies among older adults aged 65 years and above in Hong Kong and the United States reported that reduced grip strength was associated with shorter sleep duration [43] and longer sleep duration [44, 45], which is consistent with our findings. Nakakubo et al. [18] demonstrated that both short and long sleep durations were associated with slowness in both men and women, whereas Goldman et al. [46] reported that only older women with short and long sleep durations had slower gait speeds. In our study, older adults with short sleep durations had a higher rate of exhaustion, which is consistent with the results of previous studies [18, 46]. Several studies [18, 47] have also found that older adults who sleep for long durations have a higher rate of low physical activity, in line with our findings. This may be because long sleep duration is associated with poor sleep quality [48], which in turn is associated with low physical activity. The results of the present study showed that unintended weight loss was not associated with sleep duration, which is inconsistent with previous studies [45, 49]. Furthermore, previous studies [45, 49] reported that short sleep duration (< 5 h/<6 h) was associated with obesity; therefore, further research is needed to clarify this aspect. The relationship between frailty components and sleep duration differs among studies, probably due to between-study differences in participant age and ethnicity, frailty component definitions, or frailty assessment methods.

In the present study, sex-related differences were observed in the association between sleep duration and frailty. The effect of short and long sleep durations on frailty was only significant for women, and not for men; however, the underlying mechanisms are unclear. Several recent studies have addressed the possible contributors, including menopausal hormonal changes and inflammatory markers [40, 50–52]. In a prospective study conducted by Gale et al. [53], elevated inflammatory markers (CRP and fibrinogen) were predictive of incident frailty among women, but not among men. Women have higher levels of inflammatory markers [51], which could lead to catabolic processes and sarcopenia [54], thus increasing the frailty risk [54]. Therefore, there is a need to identify the effect of sex on frailty and clarify the underlying mechanisms. In addition, sex differences must be considered when developing future frailty prevention interventions.

This study has several limitations. First, we could not establish a causal relationship between sleep duration and frailty due to the cross-sectional study design. Because the association between sleep duration and frailty could be bi-directional in nature, future research should be conducted to further evaluate this causal relationship. Second, sleep duration was measured using a self-reported questionnaire. Although this is commonly applied in population-based studies, it may lead to recall bias and inaccurate results. Thus, future studies using more objective measures, such as actigraphy, are required. Third, the data used for analysis were existing secondary data from KNHANES, which were not specifically collected for sleep and frailty research. Thus, this study did not consider hypnotic drug use, pro-inflammatory factors (e.g., CRP, interleukin-6), cortisol levels, sex hormones, and various diseases (e.g., obesity-hypoventilation syndrome, obstructive apnea, heart failure, chronic obstructive pulmonary disease, and cognitive impairment), all of which can affect sleep duration and frailty. To clarify the relationship between sleep duration and frailty in older adults, we propose conducting future research that includes all of these confounding factors. Despite these limitations, our study results are meaningful because they reveal a significant association between sleep duration and frailty in a large number of participants representative of the Korean community-dwelling older adults. Furthermore, to our knowledge, this is the first Korean study to identify sex-based differences in the relationship between sleep duration and frailty.

## Conclusion

This nationwide survey of representative community-dwelling Korean older adults demonstrated that both short and long sleep durations were independently associated with frailty in women, but not in men. Therefore, the management of sleep problems among women should be prioritized and effective frailty prevention interventions should be developed accordingly. Large-scale longitudinal studies that overcome the limitations of this study would provide a more detailed understanding of the causal relationship between sleep duration and frailty.

## Data Availability

The datasets used and/or analyzed during the current study are available from the corresponding author on reasonable request.

## Abbreviations

BMI: Body mass index
CIs: Confidence intervals
CRP: C-reactive protein
KCDC: Korea Center for Disease Control
KNHANES: Korea National Health and Nutrition Examination Survey
ORs: Odds ratios
